# Value of perilesional biopsies in multiparametric magnetic resonance imaging-targeted biopsy and systematic biopsy in detection of prostate cancer: results of a prospective, non-randomized, surgeon-blinded study

**DOI:** 10.1007/s00345-024-05000-6

**Published:** 2024-05-06

**Authors:** Gregor Duwe, Melanie Schmitteckert, Maximilian Haack, Peter Sparwasser, Robert Dotzauer, Anita Thomas, Igor Tsaur, Maximilian Peter Brandt, Martin Kurosch, Rene Mager, Axel Haferkamp, Katharina Boehm, Thomas Höfner

**Affiliations:** 1 Department of Urology and Pediatric Urology, University Medical Center Johannes Gutenberg University, Langenbeckstrasse 1, 55131 Mainz, Germany; 2 Department of Urology, University Hospital and Faculty of Medicine Eberhard Karls University, Tübingen, Germany; 3 Department of Urology, University Hospital Carl Gustav-Carus, TU Dresden, Fetscherstrasse 74, 01307 Dresden, Germany; 4 Department of Urology, Ordensklinikum Linz Elisabethinen, Fadinger Strasse 1, 4020 Linz, Austria

**Keywords:** Perilesional biopsies, Prostate cancer, Clinically significant prostate cancer, Imaging-targeted prostate biopsies

## Abstract

**Purpose:**

The goal of this study is to address if detection rates of clinically significant prostate cancer (csPCa) can be increased by additional perilesional biopsies (PB) in magnetic resonance (MR)/ultrasound fusion prostate biopsy in biopsy-naïve men.

**Methods:**

This prospective, non-randomized, surgeon-blinded study was conducted between February 2020 and July 2022. Patients were included with PSA levels < 20 ng/ml and ≥ one PI-RADS lesion (grades 3–5) per prostate lobe. Prostate biopsy was performed by two urologists. The first performed the MR-fusion biopsy with 3–5 targeted biopsies (TB) and 6 PB in a standardized pattern. The second performed the systematic (12-fold) biopsy (SB) without knowledge of the MR images. Primary outcome of this study is absence or presence of csPCa (≥ ISUP grade 2) comparing TB, PB and SB, using McNemar test.

**Results:**

Analyses were performed for each PI-RADS lesion (n = 218). There was a statistically significant difference in csPC detection rate of TB + SB between PI-RADS 3, 4 and 5 lesions (18.0% vs. 42.5% vs. 82.6%, p < 0.001) and TB + PB (19.7% vs. 29.1% vs. 78.3%). Comparing only maximum ISUP grade per lesion, even SB plus TB plus PB did not detect more csPCa compared to SB plus TB (41.3% vs. 39.9%, p > 0.05).

**Conclusion:**

We present prospective study data investigating the role of perilesional biopsy in detection of prostate cancer. We detected no statistically significant difference in the detection of csPCa by the addition of PB. Therefore, we recommend continuing 12-fold bilateral SB in addition to TB.

**Supplementary Information:**

The online version contains supplementary material available at 10.1007/s00345-024-05000-6.

## Introduction

According to the European Association of Urology (EAU) guidelines, multiparametric magnetic resonance imaging (MRI) before prostate biopsy is considered the gold standard in the primary diagnosis of prostate cancer (PCa) since 2019 [1, 2]. Once MRI scans reveal a Prostate Imaging Reporting and Data System (PI-RADS) score ≥ 3, a combination of systematic (randomized) biopsies (SB) and MRI-ultrasound fusion targeted biopsies (TB) is recommended. This combined approach is based on previous multi-center randomized studies that showed an increased detection rate of clinically significant PCa (csPCa), defined as minimum International Society of Urological Pathology (ISUP) grade group 2, while decreasing rate of clinically insignificant PCa (ciPCa), defined as ISUP grade 1 [3, 4]. Currently, there is a clinically highly relevant debate to what extent systematic biopsies can be omitted and replaced by perilesional biopsies (PB) with equivalent detection rates of csPCa [5]. The key objective of previous studies and our approach in optimizing biopsy strategies is to maximize the detection of csPCa with the lowest possible number of biopsy cores and the lowest possible detection rate of ciPCa. Perilesional biopsies represent the area surrounding the PI-RADS lesion, respectively called region of interest (ROI) or “penumbra”, which is considered to be a radius approximately 5–10 mm around the ROI [6–9]. As previous studies showed that approximately 15% of csPCa could be missed without minimum 12-fold SB, it is of great interest to investigate if less PB cores might replace SB by a comparably sensitivity for csPCa [2, 6–8, 10]. Yet, the reasons for this risk of under-detecting csPCa are not sufficiently clarified. Despite the heterogeneous biology of PCa, which could lead to spreading of malignant cells throughout the prostate tissue [11, 12], other reasons discussed include underestimation of tumor volume on MRI, misinterpretation by the radiologist, or technical errors in fusion or targeting of lesions [13–17]. Previous studies investigating the role of PB support the hypothesis that PCa grows consecutively and is not fully detected by MRI imaging, which is why approximately 86% of csPCa are detected within 10 mm radius of the PI-RADS lesion [7, 16].

In conclusion, we see remarkable potential in further investigation of PB to improve primary diagnostic strategies in the detection of PCa. However, the lack in high quality study data remains to attain sufficient evidence. In the present study, we aimed to investigate the role of additional perilesional sampling to increase detection rates of csPCa in a prospective study design. We aimed to determine whether addition of six PB could eliminate the need for additional standard systematic cores without detecting less csPCA and, at the same time, not detecting more ciPCa. To our knowledge, this is the first surgeon-blinded, prospective clinical study to address this question.

## Patients and methods

### Study design and participants

**Fig. 1:**
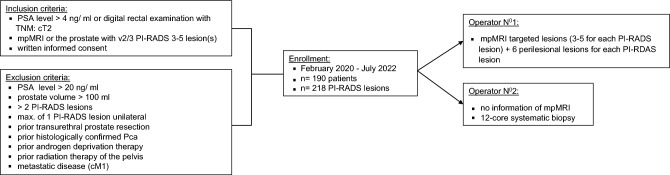
Trial profile. Abbreviation:* PSA* prostate-specific antigen, *TNM* Tumour, node and metastasis (TNM) staging, *mpMRI* multiparametric magnetic resonance imaging,* PI-RADS* Prostate Imaging Reporting and Data System

We set up a single-center (Germany), non-randomized, surgeon-blinded, prospective clinical study. Each patient served as his own control. Participants were recruited in in our academic center among patients who were referred for suspicious prostate-specific antigen (PSA) concentration or an abnormal digital rectal examination (see Fig. 1). Eligibility criteria included a serum PSA level between 4 and 20 ng/ml and a prostate volume of maximum 100 ml. We have excluded men who with any prior prostate biopsy, histologically confirmed PCa, transurethral prostate resection, androgen deprivation therapy, radiation therapy of the pelvis or any metastatic disease. The study was performed in accordance with the Declaration of Helsinki. The ethics committee of the Medical Association of Rhineland-Palatinate, Germany, approved the study (2019–14667) and all patients gave written informed consent. No commercial entity was involved in the trial.

### Procedures

Prior to study inclusion, all men underwent an MRI of the prostate within six months before the biopsy. MRI scans were performed using 1.5 Tesla or 3 Tesla scanner with or without an endorectal coil, T2-weighted imaging, diffusion-weighted imaging, and dynamic contrast enhancement. As a tertiary referral center, MRI scans were either performed in our institution or by various external institutions, representing a real-life clinical setting. Only men with a maximum of one PI-RADS score ≥ 3 lesion per lateral lobe of the prostate were included.

Based on the EAU guidelines valid at study protocol development in 2019, we only performed MRI*/* transrectal ultrasound fusion guided-targeted biopsies with local anesthesia. A preoperative application of intravenous antibiotics (ceftriaxone 2 g) one hour prior to biopsy and rectal disinfection (povidone-iodine) was performed. After the procedure, a third-generation cephalosporin was prescribed for oral administration for three more days. No infectious events were reported. Tolerability was improved by infiltration of the periprostatic plexus with local anesthesia (mecain 2%). First, a standard handheld transrectal ultrasonographically guided 12-core-systematic biopsy (SB) was performed with the urologist blinded to the MRI report. Next, the second urologist performed 3 to 5 TB depending on the size and location of the MRI lesion (individual decision of the urologist), followed by 6 systematic PB (medial, lateral, ventral, dorsal, cranial, caudal) for each targeted lesion with a maximum margin of 10 mm around the ROI. The MRI and ultrasonographic software fusion was performed using the HiVision Ascendus Ultrasound System (Hitachi Medical Systems®). This sequence was chosen to avoid operator bias by visible biopsy tracks.

### Covariates and outcome measures

Patient characteristics were obtained for age, initial PSA level, prostate volume (calculated by MRI scans), clinical stage at digital rectal examination. Radiological features included PI-RADS score, maximum lesion diameter as well as volume, and of capsule infiltration. The biopsy specific information included the International Society of Urological Pathology (ISUP) grade for each core and its core infiltration in percent (%). The primary outcome of the study was the detection rate of csPCa which was defined as an ISUP grade ≥ 2 comparing standard biopsy strategy of SB plus TB with the addition of PB to the standard protocol (SB plus TB). Secondary outcome measures of interest were the detection rate of ciPCa, defined as ISUP grade = 1, comparing standard biopsy strategy of SB plus TB with the addition of PB to the standard protocol (SB plus TB).

### Statistical analysis

We presented our outcomes according to the standards of reporting for targeted biopsy guidelines of 2013 [18]. Continuous variables are presented as mean ± standard deviation (SD) or medians ± interquartile range (IQR 25–75) in accordance with the data contribution. The cancer detection rate for csPCA was calculated by diving the number of patients diagnosed with csPCa by the total number of participants. A chi-square test was used to compare frequencies. Next, we compared the proportions of the descriptive results, stratified by the PI-RADS score 3–5, using the McNemar test with continuity correction. All tests were 2-tailed with *p* < 0.05 considered statistically significant. Statistical analysis was performed using IBM SPSS Statistics Version 27 (Armonk, NY: IBM Corp.). The Mcnemar tests were performed with RStudio Version 2022.12.0 + 353, package ‘compareGroups’ version 4.61 and package ‘stats’ version 4.2.2.

## Results

### Patients’ baseline characteristics

From February 2020 to July 2022, we consecutively enrolled 190 patients of which only 28 patients had two PI-RADS lesions which were analysed separately (Fig. 1). The patients baseline characteristics can be seen in Supplementary Table 1: The median (IQR) age at time of biopsy was 66 years (60;70), median prostate volume was 50.0 ml (35.45;65.00) and median PSA level prior to biopsy was 6.3 ng/ml (5.0;9.0). In total, 61 of the index lesions (28%) were classified as PI-RADS 3, followed by 134 lesions (61.5%) as PI-RADS 4 and 23 lesions (10.6%) as PI-RADS 5. MRI scans revealed capsule infiltration in 27 cases (12.4%). Eventually, PCa was diagnosed in 141 of cases (64.7%).

### Cancer detection rates, according to PI-RADS assessment categories and biopsy strategy groups

In total, 4.464 biopsies were taken. In a first step, we analyzed each biopsy core and compared the total number of PCa of all biopsy cores between the different groups (Supplementary Table 2). When PB were added to SB and TB (group 1 vs. 2), 33.57% more csPCa were diagnosed (423 total biopsies vs. 281, mean value (MV) per lesion: 1.94 vs. 1.29, p < 0.001) while 28.22% more ciPCa were additionally detected (528 total biopsies vs. 379, MV: 2.42 vs. 1.74, p < 0.001). Most important, TB + SB did not detect significantly more csPCa compared to TB + PB (281 total biopsies vs. 253, MV: 1.29 vs. 1.16, p = 0.111), while detecting significantly more low-risk PCa (379 vs. 257 total biopsies, MV: 1.74 compared to 1.18, p < 0.001).

Next, we compared these results on the respective maximum ISUP score per PI-RADS lesion (Supplementary Table 3) which reflects the clinically relevant definition on D’Amico risk stratification and treatment recommendation. Eventually, those higher detection rates with respect to all biopsy cores had no clinically relevant effect on the respective maximum ISUP score per PI-RADS lesion. Although, SB + TB + PB detected slightly more csPCa compared to SB + TB (41.3% vs. 39.9%,), the difference was not statistically significant (p > 0.05). The number of ciPCa was similar in both groups (23.4%). Notably, TB + PB revealed significantly less csPCa with a total number of 67 (30.7%) compared to TB + SB with a total number of 87 (39.9%), while detection rate of ciPCa was slightly lower with 49 (22.0%) compared to 51 (23.4%).

Finally, we analysed PCa detection rates, according to PI-RADS scores (Table 1). In overall comparison between PI-RADS score 3, 4 and 5 we detected statistically relevant differences in mean prostate volume between PI-RADS 3, 4 and 5 lesions (54.8 ml vs. 49.0 ml vs. 43.4 ml, p = 0.013) and mean PI-RADS lesions volume (0.38 ml vs. 0.38 ml vs. 1.86 ml, p < 0.001). First, any PCa detection rates can be compared between SB, TB and PB, demonstrating statistically relevant differences in all biopsy groups according to PI-RADS scores. Most important, we confirmed statistically higher csPCa detection rates in SB + TB (18% vs. 42.5% vs. 82.6%, p < 0.001) compared to TB + PB (19.7% vs. 29.1% vs. 78.3%, p < 0.001), according to PI-RADS scores, while SB + TB + PB only detected slightly more csPCa (23.0% vs. 44.0% vs. 82.6%, p < 0.001).

**Table 1:** Comparison of prostate cancer detection rates, according to PI-RADS assessment categories, n = 218

	3	4	5	p-value (overall)
	*n* = *61*	*n* = *134*	*n* = *23*	
Age, years (median, SD)	63.8 (5.70)	65.3 (8.10)	68.7 (6.72)	0.026
Prostate volume, ml (mean, IQR)	54.8 [43.0;71.0]	49.0 [30.8;61.2]	43.4 [32.0;58.5]	0.013
PI-RADS lesion volume, ml (mean, IQR)	0.38 [0.18;1.08]	0.38 [0.18;0.70]	1.86 [1.32;3.97]	< 0.001
Any PCa detection in TB				0.001
None	41 (67.2%)	74 (55.2%)	5 (21.7%)	
Yes	20 (32.8%)	60 (44.8%)	18 (78.3%)	
Any PCa detection in PB				< 0.001
None	45 (73.8%)	64 (47.8%)	5 (21.7%)	
Yes	14 (23.0%)	70 (52.2%)	18 (78.3%)	
‘Missing’	2 (3.28%)	0 (0.00%)	0 (0.00%)	
Any PCa detection in SB				< 0.001
None	45 (73.8%)	49 (36.6%)	2 (8.70%)	
Yes	16 (26.2%)	85 (63.4%)	21 (91.3%)	
Any PCa detection in TB + SB				< 0.001
None	37 (60.7%)	41 (30.6%)	2 (8.70%)	
Yes	24 (39.3%)	93 (69.4%)	21 (91.30%)	
Any PCa detection in TB + PB				< 0.001
None	41 (67.2%)	59 (44.0%)	3 (13.0%)	
Yes	20 (32.8%)	75 (56.0%)	20 (87.0%)	
Any PCa detection in SB + TB + PB				< 0.001
None	37 (60.7%)	41 (30.6)	2 (8.70%)	
Yes	24 (39.3%)	93 (69.4%)	21 (91.30%)	
Clinically significant Pca in SB + TB				< 0.001
None	50 (82.0%)	77 (57.5%)	4 (17.4%)	
Yes	11 (18.0%)	57 (42.5%)	19 (82.6%)	
Clinically significant Pca in TB + PB				< 0.001
None	49 (80.3%)	95 (70.9%)	5 (21.7%)	
Yes	12 (19.7%)	39 (29.1%)	18 (78.3%)	
Clinically significant Pca in SB + TB + PB				< 0.001
None	47 (77.0%)	75 (56.0%)	4 (17.4%)	
Yes	14 (23.0%)	59 (44.0%)	19 (82.6%)	

*SD* standard deviation, *IQR* interquartile range, *SB* systematic biopsies, *TB* targeted biopsies, *PB* perilesional biopsies, *PCa* prostate cancer, *ISUP* International Society of Urological Pathology, *PI-RADS* Prostate Imaging Reporting and Data System

## Discussion

We present the first prospective, surgeon-blinded, clinical trial investigating the value of additional PB for the detection of csPCa. Adding PB to the current European standard of SB plus TB significantly increase the absolute number of detected csPCa biopsy cores. However, no significant differences can be shown regarding the respective maximum ISUP grade. Previously, retrospective studies reported excellent detection rates of csPCa by combining TB plus PB (compared to SB pus TB), proposing to even omit SB in the future [6–8]. Thus, our prospective study results represent an important contribution by demonstrating non superiority of additional PB in detection of csPCa. Therefore, additional prospective randomized trials should investigate the outcome of PB in the primary diagnosis of PCa.

One possible explanation for the putative added value of PB in detection of csPCa is based on the observation that MRI-based PI-RADS lesions underdetect the true size of the tumor volume. After several preliminary works using software correlations of biopsy results and prostatectomy results [19, 20], Priester et al. have demonstrated the underdetection of MRI lesions by 11 mm in diameter and volume by threefold using whole mount pathology comparisons in 2017 [16]. After these initial findings, several retrospective clinical studies underlined the results. Brisbane et al. analyzed biopsy data of 2048 men from two large US-American centers and were able to demonstrate that 90% of csPCa was located within a radius of 10 mm around the lesions which they defined as penumbra [6]. They also described an enlarged radius based on PI-RADS lesion score from 5 mm for PI-RADS 5 lesions to 16 mm for PI-RADS 3 lesions. These findings were confirmed by Noujeim et al. who constructed patient-specific tridimensional prostate maps of 505 patients undergoing prostate biopsy [7]. They reported to detect 86% of csPCa within a 10 mm margin around the PI-RADS lesion by PB plus TB while reducing the number of biopsy cores needed by an average of six per patient. Additionally, detection of ciPCa was reduced by 19% within the 10 mm margin. Similar results are described by Hagens et al. in a retrospective analysis of 235 men in which TB plus PB detected 96.8% of csPCa while detection of ciPCa was reduced by 12.8% and mean number of biopsy cores were reduced by 5.2 [8]. Tafuri et al. demonstrated that SB can be omitted in patients with PI-RADS 5 lesions and PSA density > 0.15 ng/ml^2^ as TB alone revealed the same overall PCa detection rate [21]. These results also emphasize the importance of further risk stratifications to enhance the selection of patients which has also been proposed by several studies mentioned above, in particular in correlation with PSA density [5, 7].

A major finding of our study is that the addition of SB to TB detected significantly more additional csPCa compared to addition of PBs, which is in contrast to previous, retrospective studies [6–8]. Our results might be explained by interobserver variability in MRI analysis and possible underdetection of PI-RADS lesions, though this reflects standard practice from our point of view. In addition, prostate biopsies were performed by several urologists with different level of experience which might have an impact on fusion accuracy of MRI and ultrasound as well as on the biopsy core retrieval. While this might have decreased the quality in TB outcomes, these limitations also represent real-life situation. Moreover, higher detection rates of csPCa in the SB may be due to poor MR imaging and missing relevant lesions by MRI. High quality of MR imaging and double reading by certified radiologists in prostate MRI interpretation should be considered as standard in future, prospective trials. Lastly, we do not consider our SB outcomes to be influenced by differences in prostate volumes as median prostate volume was 50.00 ml (35.45;60.00) which is comparable to prior studies (between 45.00 [8], 48.00 [6, 7] and 52 ml [10]. However, major strength of our study is based on its prospective, non-randomized, surgeon-blinded study design that increases evidence level as compared to former retrospective data acquisitions [6–8].

Finally, different clinical interpretations of these results are reflected by the different guidelines of the international urological associations. Currently, the EAU recommends SB plus TB for each patient [1], while the American Urological Association recommends an individual approach without obligatory biopsy specifications [22]. In contrast, a much more specific guideline is presented by the PI-RADS v2 Steering Committee that recommend TB plus PB biopsies in PI-RADS 4 and 5 lesions and TB plus SB in PI-RADS 3 lesions [9].

## Conclusion

In conclusion, we present prospectively enrolled data to address the question if PB increase the detection rate of csPCa in MRI-ultrasound fusion prostate biopsies. Our key results demonstrate that the detection of maximum ISUP grade was not statistically increased by the addition of PB, while SB could not be replaced by PB in combination with TB. Therefore, we recommend continuing the 12-fold SB in addition to TB and further prospective, randomized trials.

## Supplementary Information

Below is the link to the electronic supplementary material.

Supplementary file1 (DOCX 15 KB)

Supplementary file2 (DOCX 17 KB)

Supplementary file3 (DOCX 17 KB)

## Data Availability

The data that support the findings of this study are available from the corresponding author upon reasonable request.

## Abbreviations

SD: Standard deviation
IQR: Interquartile range
SB: Systematic biopsies
TB: Targeted biopsies
PB: Perilesional biopsies
PCa: Prostate cancer
ISUP: International Society of Urological Pathology
PI-RADS: Prostate Imaging Reporting and Data System
