# Supplementary material for: Value of perilesional biopsies in multiparametric magnetic resonance imaging-targeted biopsy and systematic biopsy in detection of prostate cancer: results of a prospective, non-randomized, surgeon-blinded study
Source: World J Urol. 2024 May 6;42(1):297. doi: 10.1007/s00345-024-05000-6 (PMC11074214; doi:10.1007/s00345-024-05000-6)
Supplement: Supplementary file 1 — Supplementary file1 (DOCX 15 KB) [file 345_2024_5000_MOESM1_ESM.docx]

**Supplementary Table 1. Patients baseline characteristics, n=218**

| Characteristics (numerical) | Mean (SD) | Median (IQR) |
| --- | --- | --- |
| Age (years) | 65.26 (7.46) | 66.00 (60.00;70.00) |
| Prostata volume (ml) | 73.8 (19.95) | 50.00 (35.45;65.00) |
| iPSA (ng/ml) | 7.42 (3.52) | 6.3 (5.0;9.0) |
| **Characteristics (categorical)** | **Results** | |
|  | **n** | **%** |
| **Diagnosis of Prostata Cancer** |  |  |
| Prostate Cancer | 141 | 64.7 |
| No Prostate Cancer | 75 | 34.4 |
| **PI-RDAS v2/3 Score** |  |  |
| PI-RDAS 3 | 61 | 28 |
| PI-RDAS 4 | 134 | 61.5 |
| PI-RDAS 5 | 23 | 10.6 |
| **mpMRI Capsule Infiltration** |  |  |
| Yes | 27 | 12.4 |
| None | 191 | 87.6 |

**Abbreviation:** SD: standard deviation, IQR: interquartile range
