# Supplementary material for: Value of perilesional biopsies in multiparametric magnetic resonance imaging-targeted biopsy and systematic biopsy in detection of prostate cancer: results of a prospective, non-randomized, surgeon-blinded study
Source: World J Urol. 2024 May 6;42(1):297. doi: 10.1007/s00345-024-05000-6 (PMC11074214; doi:10.1007/s00345-024-05000-6)
Supplement: Supplementary file 2 — Supplementary file2 (DOCX 17 KB) [file 345_2024_5000_MOESM2_ESM.docx]

|  | **Group 1** | **Group 2** | **Group 3** |  |
| --- | --- | --- | --- | --- |
|  | **Systematic Biopsies (12x) + Targeted Biopsies (max. 4) + Perilesional Biopsies (6x)** | **Systematic Biopsies (12x) + Targeted Biopsies (max. 4)** | **Targeted Biopsies (max. 4) + Perilesional Biopsies (6x)** |  |
|  |  |  |  |  |
| **Number of csPCa**  **(≥ ISUP 2)** | **423** | **281** | **253** |  |
| Mean | **1.94** (SD= 3.23) | **1.29** (SD= 2.10) | 1.16 (SD= 2.17) |  |
|  | **142 more csPCa = 33.57% (14.93 related to total PCa)** | |  |  |
|  | ***p< 0.001 (group 1 vs. 2)*** | |  |  |
|  |  | ***28 less PCa = 6.62% (2.94% related to total PCa)*** | |  |
|  |  | *p= 0.111 (group 2 vs. 3)* | |  |
| **Number of ciPCa**  **(= ISUP 1)** | **528** | **379** | **257** |  |
| Mean | 2.42 (SD= 3.29) | 1.74 (SD= 2.36) | 1.18 (SD= 1.83) |  |
|  | **149 more insg. PCa = 28.22% (15.67% related to total PCa)** | |  |  |
|  | ***p< 0.001 (group 1 vs. 2)*** | |  |  |
|  |  | ***122 less ciPCa = 23.12% (12.83% related to total PCa)*** | |  |
|  |  | ***p< 0.001 (group 2 vs. 3)*** | |  |
|  |  |  | |  |

**Supplementary** **Table 2. Comparison of prostate cancer detection rates in absolute numbers of positive biopsy cores,** **according to biopsy strategy groups, n= 218**

**Abbreviation:** PCa: prostate cancer, csPCa: clinically significant prostate cancer, ciPCa: clinically insignificant prostate cancer, SD: standard deviation, ISUP: International Society of Urological Pathology
