# Supplementary material for: Value of perilesional biopsies in multiparametric magnetic resonance imaging-targeted biopsy and systematic biopsy in detection of prostate cancer: results of a prospective, non-randomized, surgeon-blinded study
Source: World J Urol. 2024 May 6;42(1):297. doi: 10.1007/s00345-024-05000-6 (PMC11074214; doi:10.1007/s00345-024-05000-6)
Supplement: Supplementary file 3 — Supplementary file3 (DOCX 17 KB) [file 345_2024_5000_MOESM3_ESM.docx]

**Supplementar**y **Table 3. Comparison of prostate cancer detection rates of maximum ISUP grade group, according to biopsy strategy groups, n= 218**

|  | **Targeted Biopsies (max. 4) + Systematic Biopsies (12x) + Perilesional Biopsies (6x)** | **Targeted Biopsies (max. 4) + Systematic Biopsies (12x)** | **Targeted Biopsies (max. 4) + Perilesional Biopsies (6x)** |  |
| --- | --- | --- | --- | --- |
|  |  |  |  |  |
| **Biopsy Ouctome - no. (%)** |  | |  |  |
| Benign tissue | 77 (35.2) | 80 (36.7) | 103 (47.2) |  |
| ISUP Grade |  |  |  |  |
| ISUP 1 | 51 (23.4) | 51 (23.4) | 48 (22.0) |  |
| ISUP 2 | 39 (17.9) | 44 (20.2) | 34 (15.6) |  |
| ISUP 3 | 30 (13.8) | 27 (12.4) | 17 (7.8) |  |
| ISUP 4 | 19 (8.7) | 16 (7.3) | 14 (6.4) |  |
| ISUP 5 | 2 (0.9) | 0 (0) | 2 (0.9) |  |
| **Clinically significant cancer - no. (%)** | **90 (41.3)** | **87 (39.9)** | **67 (30.7)** |  |
| **Clinically insignificant cancer - no. (%)** | **51 (23.4)** | **51 (23.4)** | **48 (22.0)** |  |
| **PI-RADS v2/3 Score 3** |  |  |  |  |
| Benign tissue - no. (%) | 37 (60.7) | 37 (60.7) | 41 (67.2) |  |
| Clinically significant cancer - no. (%) | 12 (19.7) | 11 (18.0) | 9 (16.4) |  |
| Clinically insignificant cancer - no. (%) | 12 (19.7) | 13 (21.3) | 10 (16.4) |  |
| **PI-RADS v2/3 Score 4** |  |  |  |  |
| Benign tissue - no. (%) | 38 (28.4) | 41 (30.6) | 59 (44.0) |  |
| Clinically significant cancer - no. (%) | 59 (44.0) | 57 (42.5) | 39 (29.2) |  |
| Clinically insignificant cancer - no. (%) | 37 (27.6) | 36 (26.9) | 36 (26.9) |  |
| **PI-RADS v2/3 Score 5** |  |  |  |  |
| Benign tissue - no. (%) | 2 (8.7) | 2 (8.7) | 3 (13.0) |  |
| Clinically significant cancer - no. (%) | 19 (82.6) | 19 (82.6) | 18 (78.2) |  |
| Clinically insignificant cancer - no. (%) | 2 (8.7) | 2 (8.7) | 2 (8.7) |  |

**Abbreviation:** ISUP: International Society of Urological Pathology
